# Dietary Pattern Accompanied with a High Food Variety Score Is Negatively Associated with Frailty in Older Adults

**DOI:** 10.3390/nu13093164

**Published:** 2021-09-10

**Authors:** Won Jang, Yoonjin Shin, Yangha Kim

**Affiliations:** 1 Department of Nutritional Science and Food Management, Ewha Womans University, Seoul 03760, Korea; jangwon1011@naver.com (W.J.); yjin19@hotmail.com (Y.S.); 2 Graduate Program in System Health Science and Engineering, Ewha Womans Universty, Seoul 03760, Korea

**Keywords:** frailty, dietary pattern, food variety score

## Abstract

Proper nutrition is a modifiable factor in preventing frailty. This study was conducted to identify the association between dietary patterns and frailty in the older adult population. The cross-sectional analysis was performed on 4632 subjects aged ≥65 years enrolled in the Korea National Health and Nutrition Examination Survey from 2014–2018. Food variety score (FVS) was defined as the number of foods items consumed over a day. Three dietary patterns were identified using factor analysis: “white rice and salted vegetables,” “vegetables, oils, and fish,” and “noodles and meat.” The higher “white rice and salted vegetables” pattern score was related to significantly lower FVS, whereas higher “vegetables, oils, and fish” and “noodles and meat” pattern scores were associated with a higher FVS. Participants with higher FVS showed a low risk of frailty (odds ratio (OR) (95% confidence interval, CI) = 0.44 (0.31–0.61), *p*-trend = 0.0001) than those with lower FVS. Moreover, the “vegetables, oils, and fish” pattern score was significantly associated with a low risk of frailty (OR (95% CI) = 0.55 (0.40–0.75), *p*-trend = 0.0002). These results suggested that consuming a dietary pattern based on vegetables, oils, and fish with high FVS might ameliorate frailty in older adults.

## 1. Introduction

Given the rapid aging of the world’s population, the prevention of frailty is becoming more important than ever. Frailty is a geriatric syndrome characterized by a reduced physical and psychological function and a decline in the ability to maintain homeostasis [1]. Frailty in older adults is a risk factor for falls, morbidity, disability, and even mortality [2] and is often viewed as a major challenge for medical and health care services [3]. Thus, preventing frailty can help reduce medical and related costs and address the challenges of successful aging.

Nutritional status is considered one of the modifiable risk factors for frailty. It is well known that inadequate protein and micronutrients can contribute to frailty [4]. However, understanding the nutrition–health interface requires shifting the focus from individual nutrients toward food-based approaches. In this regard, investigating the impact of dietary patterns on frailty may be more useful than analyzing the effects of a single nutrient in establishing a frailty prevention strategy. In Western countries, adherence to a Mediterranean diet has been associated with a reduced incidence of frailty among older people [4,5,6], but whether there is an association between frailty and dietary patterns other than the Mediterranean diet is still unknown [7]. It is difficult to compare the results of dietary patterns between populations because dietary patterns are strongly related to the study population’s diet.

Therefore, it would be interesting to understand the relationship between dietary patterns and frailty and the impact of dietary quality to identify the most appropriate diets for decreasing frailty prevalence. The food variety score (FVS) is a simple count of food items and has been proven to be a useful indicator of the nutritional adequacy of the diet [8]. Consuming a wide variety of food groups increases the chances of providing the various nutrients and phytochemicals needed for optimal health, which could reduce the risk of frailty [9]. In contrast, inadequate nutrient intake that accompanies poor dietary variety causes oxidative stress [10] and inflammatory reactions [11], contributing to the risk of frailty. In some studies, frailty decreased as a result of high overall diet quality [12], characterized by increased consumption of fruits and vegetables [13] and optimal intakes of antioxidant nutrients [14].

Dietary variation is important for health maintenance and disease prevention in older adults. Many prior studies have reported the association between healthy dietary patterns and frailty, but most of them have been focused on Western populations [5,6,7]. Very few studies have been conducted with Asian population [8,10,15], and the results are not consistent. Thus, in-depth research on the association between dietary factors and frailty is highly needed. Therefore, this study aimed to identify dietary patterns related to frailty in a larger sample of older Korean adults. Moreover, we investigate not only dietary patterns but also dietary variety that is comprehensively available in various food culture as an indicator of dietary factors related to frailty.

## 2. Materials and Methods

### 2.1. Data Collection

This study used the data from the 2014–2018 Korea National Health and Nutrition Examination Survey (KNHANES), which included KNHANES VI (2013–2015) and KNHANES VII (2016–2018), conducted by the Korea Centers for Disease Control and Prevention (KCDC). The KNHANES is an ongoing cross-sectional survey designed to use complex, multistage, stratified, and probability cluster sampling to obtain nationally representative estimates [16]. The investigation included a health questionnaire, health examination, and nutrition surveys. The Institutional Review Board (IRB) of the KCDC approved this study (2013-07CON-03-4C, 2013-12EXP-03-5C, 2018-01-03-P-A). Detailed information about the data and survey is available on the KNHANES website (http://knhanes.cdc.go.kr accessed on 9 September 2021).

### 2.2. Subjects

The participants in the 2014–2018 survey totaled 39,199. The present analysis was limited to adults aged 65 or older who completed the survey (*n* = 7166). Participants with incomplete data on frailty classification were excluded (*n* = 1229). Those with missing dietary intake data, having energy intakes below 500 kcal and over 5000 kcal, and unusual intake on the previous day were also excluded (*n* = 1232). In addition, we excluded subjects with missing data on other covariates, such as sociodemographic information, smoking, and alcohol consumption (*n* = 73). Thus, 4632 subjects were included in the study.

### 2.3. Frailty Classification

Frailty was measured using a slight modification of the five criteria for the frailty phenotype developed by Fried et al. [17]: (1) unintentional weight loss (self-reported unintentional weight loss in the last year of >3 kg) [18], (2) exhaustion (if self-perception of stress is extremely high, it is considered to be emotional/physical exhaustion) [19], (3) weakness (handgrip strength <26 kg for men and <18 kg for women based on the Asian Working Group criteria for sarcopenia) [20], (4) walking difficulties (if the subjects responded to the mobility question of the European Quality of Life 5-Dimensions (EuroQoL-5D) questionnaire that walking was difficult, it was classified as walking difficulties) [21], and (5) low physical activity (physical activity was measured using the Global Physical Activity Questionnaire (GPAQ) developed by the World Health Organization (WHO) and was classified as low physical activity when recreational activity was <2 h per week) [22]. Participants were classified as robust if they fulfilled none of the criteria, pre-frail if they fulfilled one or two criteria, and frail if they fulfilled three or more criteria.

### 2.4. Dietary Assessment

Dietary intake information was obtained from a nutrition survey of the KNHANES using the 24 h recall method [16]. Skilled and well-trained dietary interviewers conducted the 24 h recall by face-to-face interview. The participants reported all the food and beverage that was consumed the previous day, including food name, types of ingredients, and amount of food intake per meal. For the analysis of dietary patterns, food items from the 24 h recall data were integrated into 18 food groups based on similarities. The grains and grain products group accounted for nearly half of the daily energy intake, so this food group was further divided into white rice, grains, noodles and dumplings, flour, bread and rice cakes, and pizza and hamburgers. Salted vegetables, including kimchi, were separated from other raw vegetables because, as a traditional fermented food in Korea, it has a high frequency of consumption and contains high sodium. Beverages were divided into alcohol, coffee and tea, and sugar-sweetened beverages. Our final analysis included a total of 24 food groups. The difference in weight between solid and liquid foods was corrected by representing the food groups as a percentage of energy.

To assess diet quality, the overall FVS was adopted. FVS was calculated by the simple count of the number of food items consumed by each subject during the last 24 h [23]. If the main ingredients were the same, they were classified as the same food items even if prepared by different cooking methods. The amount of food consumed and the frequency of consumption were not taken into account.

### 2.5. Assessment of Other Variables

Information on demographic and socioeconomic characteristics, including age, body mass index (BMI; kg/m^2^), living status (living alone, living with others), residential area (urban, rural), education level (≤elementary school, ≥middle school), household income level (≤the lowest quartile, ≥middle–low), smoking status (current smoker or non-current smoker), high-risk alcohol consumption (yes or no), and comorbidity (whether subjects suffered from three or more simultaneous diseases diagnosed by a doctor), was obtained using a general questionnaire and health interview questionnaire.

### 2.6. Statistical Analysis

All statistical analyses were performed using SAS software version 9.4 (SAS Institute, Inc., Cary, NC, USA). Due to the complex sampling design of the KNHANES study, sample weights, stratifying variables (*k* strata), and primary sampling units were included in our analysis. The dietary pattern was derived using factor analysis with the FACTOR procedure and VARIMAX rotation function that maintains uncorrelated factors and increases interpretability. Eigenvalues, scree plot, and interpretability ability were considered in deciding the number of factors. Significance was given to the food group whose factor load value exceeded 0.25 or −0.25. Scores of the individual dietary patterns of the whole population were categorized into tertiles and used for comparison of FVS, nutrient intake, and other general characteristics. Differences in the distribution of characteristics between tertiles of dietary pattern scores were analyzed using the SURVEY FREQ procedure for categorical variables or the SURVEY MEAN procedure for continuous variables. Significant differences between tertiles of dietary patterns were determined using the χ^2^ test or a general linear model (Scheffe’s test of multiple comparisons). Multinomial SURVEYLOGISTIC analysis was performed to estimate the odds ratios (ORs) and 95% confidence intervals (CIs) for frailty (robust vs. pre-frail vs. frail, with robust as the reference) across tertiles of dietary pattern scores and FVS. Adjustments were performed for potential confounding variables, selected based on the prior knowledge from the scientific literature and whether they are related to the independent and dependent variables. Confounders included age, gender, BMI, residential area, family income, education level, smoking status, high-risk alcohol consumption, total energy intake, and comorbidity and there was no significant multicollinearity among these variables. All reported probability tests were two-sided, with a *p*-value < 0.05 considered statistically significant.

## 3. Results

### 3.1. General Characteristics of Study Subjects

The general characteristics of the study population are presented in Table 1. Of the 2184 males (48.8%) and 2448 females (51.2%) included in the study, 17.5% lived alone, 21.6% were rural residents, and 78.4% were urban residents. The average age of the study subjects was 72.5 ± 0.1 years. More than half of the subjects had education levels below elementary school (56.2%), and 44.9% had income levels below the lowest quartile. Comorbidity, defined as having more than three diseases simultaneously, was seen in 19.7% of the subjects.

### 3.2. Dietary Patterns in the Study Population

Table 2 gives the three dietary patterns identified by factor analysis. Pattern 1 showed the highest factor loadings for white rice and kimchi and salted vegetables and negative loadings for flour, pizza, snacks, and fruits. We named Pattern 1 “white rice and salted vegetables.” Pattern 2 had the highest factor loadings for non-salted vegetables, seasonings, oils, and fish and shellfish, so we described Pattern 2 as “vegetables, oils, and fish.” Pattern 3 had high factor loadings for noodles and dumplings, meat, alcohol, and coffee and tea, and negative loadings for fruits and non-salted vegetables. We named Pattern 3 “noodles and meat.” These three patterns accounted for 19.5% of the total variance in food intakes.

### 3.3. Comparison of General Characteristics by Tertiles of Dietary Pattern Scores

The subjects’ general characteristics across the tertiles of the dietary pattern scores are summarized in Table 3. Subjects in the highest tertile of the “white rice and salted vegetables” dietary pattern tended to be male, older, rural residents of high-risk alcohol consumption with a lower education level and lower family income level than those in the lowest tertile. Meanwhile, subjects in the highest tertile of the “vegetables, oils, and fish” pattern tended to be younger, living alone, urban residents, and highly educated, with higher family income and lower comorbidity than their lowest tertile counterparts. Lastly, the highest tertile of the “noodles and meat” pattern was associated with subjects with high-risk alcohol consumption that were less educated and more likely to smoke than those in the lowest tertile of this dietary pattern (Table 3).

### 3.4. FVS and Nutrient Intakes across Tertiles of Dietary Pattern Scores

Table 4 lists the age- and sex-adjusted mean values for FVS and nutrient intakes across the tertiles of the dietary pattern scores. Both the “vegetables, oils, and fish” and the “noodles and meat” patterns showed a significant positive trend of FVS. On the contrary, the “white rice and salted vegetables” pattern showed a significant negative tendency for FVS. The “white rice and salted vegetables” pattern showed a significant negative association with energy, energy from protein, energy from fat, and intake of nutrients, such as fiber, calcium, phosphorus, potassium, thiamin, riboflavin, vitamin C, ω-3/-6 polyunsaturated fatty acids (PUFA), flavonoids, and carotenoids. Conformability to the “vegetables, oils, and fish” pattern was significantly positively related to energy, energy from protein, energy from fat, and intake of nutrients, such as fiber, calcium, iron, sodium, potassium, thiamin, riboflavin, niacin, vitamin C, ω-3/-6 PUFA, flavonoids, and carotenoids. In the “noodles and meat” pattern, there was a significantly positive tendency to consume energy and iron but a negative tendency for other nutrients.

### 3.5. Association of Frailty with Dietary Pattern Scores Considering FVS

**Figure 1:**
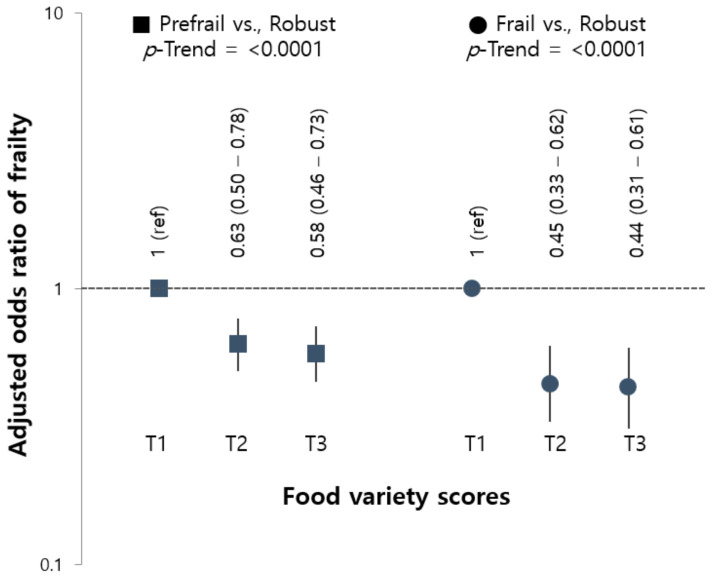
Adjusted ORs (95% CI) for pre-frailty and frailty according to the tertile of food variety scores. Ref.: reference category; OR: odds ratio; CI: confidence interval. Data were calculated using the multinomial SURVEYLOGISTIC model. ORs (95% CIs) were adjusted for gender, age, BMI, living status, residential area, family income, education level, smoking status, high-risk alcohol consumption, comorbidity, and energy intake.

The prevalence of frail and pre-frail in this study was 11.9% (*n* = 572) and 62.5% (*n* = 2945), respectively. From the results of the multinomial logistic analysis for the association of frailty with the tertile of FVS (Figure 1), frailty was inversely associated with each tertile of FVS. The OR of pre-frail and frail was significantly lower in the highest tertile of FVS (OR (95% CI) = 0.44 (0.31–0.61), *p*-trend < 0.0001) compared to the lowest tertile.

**Figure 2:**
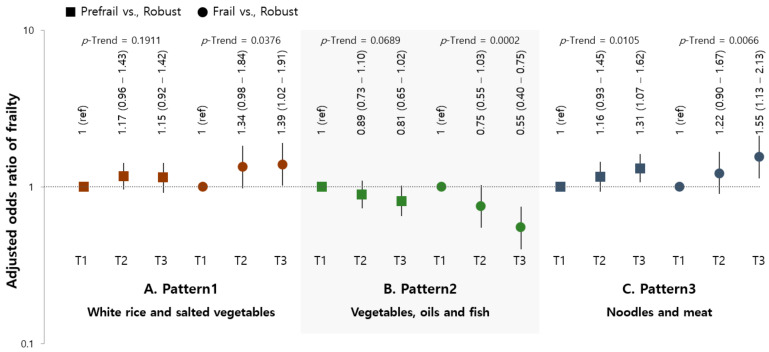
Adjusted ORs (95% CI) for pre-frailty and frailty according to the tertile of dietary pattern scores. (**A**) “White rice and salted vegetables”; (**B**) “Vegetables, oils, and fish”; (**C**) “Noodles and meat.” Ref.: reference category; OR: odds ratio; CI: confidence interval. Data were calculated using the multinomial SURVEYLOGISTIC model. ORs (95% CIs) were adjusted for gender, age, BMI, living status, residential area, family income, education level, smoking status, high-risk alcohol consumption, comorbidity, and energy intake.

The results of the multinomial logistic analysis for the association between frailty and dietary pattern score are presented in Figure 2. The highest tertile of the “vegetables, oils, and fish” pattern was significantly inversely associated with frailty (OR (95% CI) = 0.55 (0.40–0.75), *p*-trend = 0.0002). The ORs (95% CI) of frailty for those in the highest tertile compared to the lowest tertile of pattern scores were 1.39 (1.02–1.91) for the “white rice and salted vegetables” pattern (*p*-trend = 0.0376) and 1.55 (1.13–2.13) for the “noodles and meat” pattern (*p*-trend = 0.0066).

## 4. Discussion

This study was conducted to identify the relationship of dietary patterns with frailty considering food variety. A greater food variety was significantly associated with lower odds of frailty. Three major dietary patterns were identified in this study of older Korean adults: “white rice and salted vegetables,” “vegetables, oils, and fish,” and “noodles and meat.” Among these patterns, “vegetables, oils, and fish” was associated positively with FVS and showed an inverse relationship with the risk of frailty.

Assessing the relationship between individual nutrients and frailty may not take into consideration the interactions between nutrients. An increasing number of investigations in recent years has evaluated the association between dietary patterns and frailty. As mentioned above, some studies in Western countries have reported that adherence to Mediterranean dietary patterns protects against frailty [4,5]. In this current study of the specific dietary pattern of Koreans, we found that the highest tertile of the “vegetables, oils, and fish” pattern was associated with a low prevalence of frailty. A prospective study of older Spanish adults [6] found that a “prudent” dietary pattern (characterized by a high intake of olive oil and vegetables) was inversely associated with frailty incidence. Furthermore, in a study of Taiwanese older adults, a reduced prevalence of frailty was observed in those with a dietary pattern high in ω-3-rich deep-sea fish, phytonutrient-rich plant foods, and other protein-rich foods, such as shellfish and milk [24]. In another study involving a large cohort of older European subjects, high consumption of fruits and vegetables was associated with a reduced frailty risk [5]. A sufficient intake of fish or oils rich in *ω*-3 and vegetables rich in antioxidants and phytonutrients could have the effect of preventing frailty through various mechanisms, including anti-oxidative, anti-inflammatory, and muscle decomposition prevention. Similar to our research, one study of the Korean population reported that dietary patterns with high consumption of meat, fish, and vegetables lower the risk of pre-frailty. It suggests a potentially protective effect against frailty of a protein-rich and vegetable-rich food pattern [15]. Dietary patterns are analyzed based on the diet of the study reference population, so the dietary patterns extracted from each study may be different. In the present study, a dietary pattern of “white rice and salted vegetables” or “noodles and meat” was associated with an increased risk of frailty. This outcome might also be linked to oxidative stress and inflammation because high intakes of carbohydrates, sodium, red meat, and *N*-nitroso compounds found in processed meat products are related to oxidative stress and inflammation [25,26].

In a prospective investigation of older Chinese people there was no association between a dietary pattern including a “vegetable–fruit” pattern and the incidence of frailty [7]. The FVS refers to the total number of different food items consumed individually over a particular time. It is the main measure used to assess the overall diet and has been associated with the nutrient adequacy ratio of the nutrients and diet quality [8]. A previous study reported that the FVS could reflect the overall dietary quality and is related to the health status of Korean adults [27]. In this current study, considering both dietary pattern and food diversity, the higher the compliance with the “vegetable, oils, and fish” pattern, the higher the FVS and the lower the prevalence of frailty. On the contrary, the “white rice and salted vegetables” pattern was inversely associated with FVS and an increased risk of frailty. Furthermore, we found that a lower diet diversity, indicated by a significantly low FVS, was also associated with frailty. This finding is consistent with another study that found frailty was intimately related to low dietary diversity in older adults [9]. Low food variety and a narrow range of food choices may result in an inadequate intake of micronutrients and phytochemicals [28]. Antioxidant nutrients could reduce the risk of frailty through different biological pathways, such as oxidative stress [10] and inflammation [11]. Antioxidant nutrients have been shown to protect against oxidative stresses that may cause muscle atrophy and loss of muscle fibers [10]. Inflammation is an inevitable reaction to the aging process and plays an important role in frailty pathogenesis by influencing key components of the frailty syndrome. Moreover, increased pro-inflammatory cytokines and interleukin-6 have been associated with slow walking speed and reduced muscle strength [29]. High levels of C-reactive protein have also been associated with frailty [30].

In our study, we found no detail confirming whether frailty was related to individual macronutrients, such as carbohydrates, proteins, and fats. Protein intake has been a major focus of literature studies evaluating specific nutrients related to frailty because of the progressive loss of muscle mass and strength with aging. Amino acids stimulate muscle protein synthesis. However, previous studies on protein intake and frailty showed some-what contradictory results [31,32,33,34]. Bartali et al. [31] reported an association of low protein intake and frailty after adjusting for energy intake. Kobayashi et al. [32] showed that increased total protein intake was associated with a decreased prevalence of frailty among older Japanese women. However, our research did not follow these results. As with our findings, Schoufour et al. [33] and Shikany et al. [34] did not observe an association between frailty and energy-adjusted protein intake. Although the overall quality of meals and dietary patterns are more important than single nutrients, the importance of protein intake in older adults should not be overlooked in relation to muscle strength, the main cause of aging. In our study, the positive association of the “vegetables, oils, and fish” pattern, which was inversely related to frailty, and the protein-energy intake, might provide some evidence to support the relationship between frailty and protein. Detailed research on frailty and proteins is needed, considering not only amount of protein intake but also the source of protein.

The present study has some limitations. First, because of the cross-sectional nature of KNHANES, we could not define the causality of frailty and dietary factors. Further investigation is warranted to identify the causal relationship. Second, as a retrospective study, we developed a modified frailty index using the variables investigated in KNHANES to analyze the association between frailty with nutritional factors. In addition to the phenotype of frailty, there may be several other operations and definitions of frailty. Third, our dietary data were derived from a single 24 h dietary recall, which may not be sufficient to estimate usual dietary intake. However, only minor variations were observed between a single-day (24 h) dietary recall and data obtained over 2–10 days (3.9% for energy, within 10% for macronutrients and micronutrients) in the 2009 KNHANES [35]. Nevertheless, to the best of our knowledge, this current study is the first to report that a dietary pattern of “vegetables, oils, and fish” that includes diverse food items might be a therapeutic approach to decreasing frailty among older adults, based on a nationally representative population.

## 5. Conclusions

Our findings revealed that dietary pattern characterized by a high intake of vegetables, oils, and fish with a wide variety of foods might decrease frailty among older adults. To prevent frailty in older adults, encouraging the consumption of various kinds of food based on an increased intake of vegetables, oils, and fish may be one of the most straightforward and effective public nutrition strategies for the aging population. Future longitudinal study may be promising to confirm the causal association between dietary pattern and the risk of frailty.

## Figures and Tables

**Table 1:** General characteristics of the study subjects.

Variables	Total (*n* = 4632)
Age (years, mean ± SE)	72.5 ± 0.1
Age range	
65–69	1538 (33.6)
70–79	2411 (51.9)
≥80	683 (14.5)
Gender	
Male	2184 (48.8)
Female	2448 (51.2)
Living status	
Living alone	990 (17.5)
Living with others	3642 (82.5)
Residence	
Rural	1300 (21.6)
Urban	3332 (78.4)
Education	
≤Elementary school	2708 (56.2
≥Middle school	1924 (43.8)
Family income	
≤The lowest quartile	2164 (44.9)
≥Middle–low	2468 (55.1)
Current smoking status	
Current smoker	439 (9.6)
Non-current smoker	4193 (90.4)
High-risk alcohol consumption	
Yes	187 (4.1)
No	4444 (95.9)
Body mass index (kg/m^2^, mean ± SE)	24.0 ± 0.1
Body mass index range	
<18.5	120 (2.6)
18.5–24.9	2833 (61.7)
≥25.0	1679 (35.7)
Comorbidity	
Yes	925 (19.7)
No	3707 (80.3)

Values are presented as mean ± standard error (SE) or *n* (%).

**Table 2:** Factor loading matrix for the three dietary patterns of older Korean adults.

Food Group	Pattern 1	Pattern 2	Pattern 3
	White Rice and Kimchi	Vegetables, Oils, and Fish	Noodles and Meat
White rice	0.83902		−0.27383
Grains			
Noodles and dumplings	−0.2601		0.54391
Flour, bread, and rice cakes	−0.41547		
Hamburgers, pizza, and snacks			
Potatoes	−0.30472		
Sweets			
Beans			
Nuts	−0.3071		−0.33496
Non-salted vegetables		0.61885	
Kimchi and salted vegetables	0.32283		
Mushrooms			
Fruits	−0.40244		−0.34568
Meats			0.42253
Processed meats			
Eggs	−0.32273		
Fish and shellfish		0.47125	
Seaweed			
Milk and dairy products	−0.38096		
Oils		0.51915	
Alcohol		0.27234	0.38311
Coffee and tea			0.37365
Sugar-sweetened beverages			
Seasonings		0.53593	
Variance explained (%)	8.15%	5.85%	5.53%

Factor loading values < |0.25| were excluded for simplicity. The patterns were derived based on the energy contribution ratio of food groups by factor analysis.

**Table 3:** General characteristics across tertiles of dietary pattern scores.

Variables	Dietary Pattern Score											
	Pattern 1 White Rice and Salted Vegetables				Pattern 2 Vegetables, Oils, and Fish				Pattern 3 Noodles and Meat			
	Tertile 1 (Lowest) (*n* = 1544)	Tertile 2 (Middle) (*n* = 1544)	Tertile 3 (Highest) (*n* = 1544)	*p*-Value	Tertile 1 (Lowest) (*n* = 1544)	Tertile 2 (Middle) (*n* = 1544)	Tertile 3 (Highest) (*n* = 1544)	*p*-Value	Tertile 1 (Lowest) (*n* = 1544)	Tertile 2 (Middle) (*n* = 1544)	Tertile 3 (Highest) (*n* = 1544)	*p*-Value
Age (years, mean ± SE)	71.6 ± 0.2	72.5 ± 0.2	73.6 ± 0.1	<0.0001	73.1 ± 0.1	72.7 ± 0.2	71.8 ± 0.2	<0.0001	72.2 ± 0.2	72.9 ± 0.1	72.5 ± 0.2	0.0037
Age range (years)												
65–69	622 (41.3)	518 (33.6)	398 (25.6)	<0.0001	415 (27.9)	516 (32.7)	607 (39.9)	<0.0001	548 (36.3)	487 (30.8)	503 (33.7)	0.0448
70–79	745 (47.1)	811 (53.1)	855 (55.7)		850 (55.4)	807 (52.3)	754 (48.2)		796 (51.0)	809 (53.8)	806 (50.9)	
80≤	177 (11.5)	215 (13.3)	291 (18.7)		279 (16.7)	221 (15.0)	183 (11.9)		200 (12.7)	248 (15.4)	235 (15.4)	
Gender (male, %)	612 (41.2)	782 (52.5)	790 (52.8)	<0.0001	630 (41.0)	736 (50.4)	818 (54.7)	<0.0001	581 (39.6)	710 (47.5)	893 (59.2)	<0.0001
Living status (living alone, %)	317 (17.3)	314 (16.7)	359 (18.5)	0.4475	414 (21.9)	276 (14.8)	300 (16.1)	<0.0001	343 (18.0)	326 (17.3)	321 (17.3)	0.8580
Residence (rural, %)	302 (15.0)	442 (21.4)	556 (28.7)	<0.0001	495 (24.2)	419 (21.7)	386 (19.2)	0.0147	393 (19.8)	453 (22.4)	454 (22.7)	0.1996
Education (≤elementary school, %)	707 (43.5)	910 (56.5)	1091 (68.9)	<0.0001	1042 (64.9)	896 (57.1)	770 (46.8)	<0.0001	895 (54.4)	951 (60.3)	862 (53.7)	0.0031
Family income level (≤low, %)	549 (34.9)	715 (44.9)	900 (55.3)	<0.0001	851 (52.0)	695 (43.1)	618 (39.9)	<0.0001	721 (44.0)	750 (47.5)	693 (43.2)	0.0974
Current smoker (%)	102 (2.3)	145 (9.8)	192 (12.3)	<0.0001	135 (9.0)	138 (8.8)	166 (11.0)	0.1539	94 (6.0)	127 (8.8)	218 (14.0)	<0.0001
High-risk alcohol consumption (%)	42 (2.6)	61 (3.8)	84 (5.7)	0.0002	38 (2.4)	47 (3.1)	102 (6.6)	<0.0001	38 (2.3)	43 (2.8)	106 (7.0)	<0.0001
Body mass index (kg/m^2^, mean ± SE)	24.2 ± 0.1	24.1 ± 0.1	23.9 ± 0.1	0.1164	24.0 ± 0.1	24.0 ± 0.1	24.1 ± 0.1	0.5946	24.0 ± 0.1	24.0 ± 0.1	24.1 ± 0.1	0.6819
Body mass index range (kg/m^2^)												
<18.5	28 (1.8)	35 (2.2)	57 (3.9)	0.0157	53 (3.8)	37 (2.4)	30 (1.7)	0.0222	35 (2.1)	43 (3.1)	42 (2.7)	0.5585
18.5–24.9	934 (60.9)	949 (61.9)	950 (62.2)		923 (59.7)	963 (63.2)	947 (62.1)		963 (63.1)	930 (60.7)	940 (61.3)	
≥25.0	582 (37.3)	560 (35.8)	537 (33.9)		568 (36.5)	544 (34.4)	567 (36.2)		546 (34.8)	571 (36.2)	562 (36.0)	
Comorbidity (%)	334 (21.2)	307 (20.1)	284 (17.6)	0.0692	324 (20.6)	328 (21.4)	273 (17.1)	0.0192	340 (21.2)	296 (18.9)	289 (19.0)	0.2871

Values are presented as mean ± standard error (SE) or n (%). All p-values were determined by the chi-square test and general linear model.

**Table 4:** Age- and sex-adjusted mean daily energy and nutrient intake across tertiles of food variety scores and dietary pattern scores.

	Dietary Pattern Score											
	Pattern 1 White Rice and Salted Vegetables				Pattern 2 Vegetables, Oils, and Fish				Pattern 3 Noodles and Meat			
	Tertile 1	Tertile 2	Tertile 3	*p*-Trend	Tertile 1	Tertile 2	Tertile 3	*p*-Trend	Tertile 1	Tertile 2	Tertile 3	*p*-Trend
Food variety score	34.7 ± 0.4	32.9 ± 0.4	25.5 ± 0.4	<0.0001	24.4 ± 0.4	32.7 ± 0.4	36.0 ± 0.5	<0.0001	29.9 ± 0.4	31.4 ± 0.4	32.0 ± 0.4	0.0077
Total energy (kcal)	1803.4 ± 21.0	1722.2 ± 18.4	1548.4 ± 19.3	<0.0001	1655.0 ± 19.7	1701.7 ± 19.2	1719.9 ± 20.1	<0.0001	1659.0 ± 21.5	1648.4 ± 17.8	1771.2 ± 20.9	<0.0001
percentage from energy												
Carbohydrates (%)	68.7 ± 0.3	71.1 ± 0.3	74.8 ± 0.3	<0.0001	76.3 ± 0.3	72.2 ± 0.3	66.1 ± 0.3	<0.0001	74.5 ± 0.3	73.4 ± 0.3	66.6 ± 0.3	<0.0001
Protein (%)	13.4 ± 0.1	13.2 ± 0.1	12.1 ± 0.1	<0.0001	11.2 ± 0.1	13.1 ± 0.1	14.4 ± 0.1	<0.0001	13.1 ± 0.1	12.7 ± 0.1	13.0 ± 0.1	0.7579
Fat (%)	17.3 ± 0.2	13.4 ± 0.2	8.8 ± 0.2	<0.0001	10.6 ± 0.2	12.9 ± 0.2	16.0 ± 0.3	<0.0001	12.2 ± 0.2	11.8 ± 0.2	15.6 ± 0.2	<0.0001
Fiber (g)	29.7 ± 0.5	25.8 ± 0.4	21.0 ± 0.4	<0.0001	22.3 ± 0.5	26.2 ± 0.5	28.1 ± 0.5	<0.0001	31.2 ± 0.5	23.6 ± 0.4	21.9 ± 0.4	<0.0001
Calcium (mg)	507.7 ± 9.7	444.7 ± 9.3	365.4 ± 8.9	<0.0001	364.0 ± 6.8	433.1 ± 8.1	520.8 ± 11.7	<0.0001	515.4 ± 12.3	416.8 ± 7.7	387.6 ± 6.7	<0.0001
Phosphorus (mg)	1003.6 ± 13.1	934.9 ± 11.7	779.2 ± 11.8	<0.0001	780.2 ± 10.7	916.9 ± 11.7	1020.6 ± 14.1	<0.0001	985.4 ± 14.8	877.7 ± 10.9	857.9 ± 11.6	<0.0001
Iron (mg)	13.3 ± 0.3	13.7 ± 0.3	11.9 ± 0.2	0.2458	11.4 ± 0.2	13.0 ± 0.3	14.6 ± 0.3	<0.0001	14.6 ± 0.3	12.7 ± 0.2	11.7 ± 0.2	<0.0001
Sodium (mg)	3179.9 ± 61.9	3097.1 ± 60.8	2725.9 ± 59.1	0.4835	2465.6 ± 51.9	2913.5 ± 55.1	3615.2 ± 71.7	<0.0001	2782.3 ± 62.9	2818.5 ± 51.9	3413.0 ± 64.5	<0.0001
Potassium	3069.1 ± 47.7	2715.1 ± 34.8	2197.7 ± 34.8	<0.0001	2302.9 ± 37.6	2680.9 ± 38.5	3001.7 ± 46.8	<0.0001	3028.9 ± 49.3	2555.7 ± 36.4	2409.7 ± 37.2	<0.0001
Vitamin A (μg RAE)	341.0 ± 9.9	318.3 ± 10.8	293.2 ± 22.8	0.9197	204.6 ± 11.1	294.7 ± 8.7	450.8 ± 20.6	<0.0001	391.5 ± 21.7	292.6 ± 9.4	269.0 ± 10.6	<0.0001
Thiamin (mg)	1.3 ± 0.0	1.5 ± 0.0	1.3 ± 0.0	<0.0001	1.2 ± 0.0	1.4 ± 0.0	1.5 ± 0.0	<0.0001	1.4 ± 0.0	1.4 ± 0.0	1.3 ± 0.0	<0.0001
Riboflavin (mg)	1.4 ± 0.0	1.1 ± 0.0	0.8 ± 0.0	<0.0001	0.9 ± 0.0	1.1 ± 0.0	1.3 ± 0.0	<0.0001	1.2 ± 0.0	1.0 ± 0.0	1.1 ± 0.0	<0.0001
Niacin (mg)	11.9 ± 0.1	11.8 ± 0.2	10.1 ± 0.2	0.6320	9.6 ± 0.2	11.8 ± 0.2	13.0 ± 0.2	<0.0001	11.9 ± 0.2	10.8 ± 0.2	11.2 ± 0.2	<0.0001
Vitamin C (mg)	91.4 ± 3.4	75.9 ± 3.6	48.7 ± 1.8	<0.0001	63.2 ± 3.9	68.9 ± 2.3	84.3 ± 2.7	<0.0001	97.0 ± 4.1	64.0 ± 2.0	55.6 ± 2.3	<0.0001
ω-3 PUFA (g)	1.8 ± 0.1	1.5 ± 0.0	1.1 ± 0.1	<0.0001	0.8 ± 0.0	1.4 ± 0.0	2.2 ± 0.1	<0.0001	1.8 ± 0.1	1.4 ± 0.1	1.3 ± 0.0	<0.0001
ω-6 PUFA (g)	8.6 ± 0.2	6.2 ± 0.1	3.9 ± 0.1	<0.0001	4.4 ± 0.1	6.0 ± 0.1	8.3 ± 0.2	<0.0001	6.5 ± 0.2	5.4 ± 0.1	6.9 ± 0.2	0.8982
Flavonoids (mg)	102.3 ± 2.6	91.2 ± 2.3	75.9 ± 2.3	<0.0001	79.6 ± 2.6	91.6 ± 2.4	98.4 ± 2.7	<0.0001	114.9 ± 3.0	82.0 ± 2.0	73.0 ± 2.1	<0.0001
Carotenoids (mg)	11.6 ± 0.4	9.4 ± 0.2	6.8 ± 0.2	<0.0001	7.3 ± 0.3	98.4 ± 2.7	11.5 ± 0.4	<0.0001	11.8 ± 0.4	6.9 ± 0.2	7.0 ± 0.2	<0.0001

Values are presented as means (least-square means) ± standard error adjusted for gender and age. PUFA, polyunsaturated fatty acid.

## Data Availability

Data are available from the Korea National Health and Nutrition Examination Survey (KNHANES VI and VII), conducted by the Korea Centers for Disease Control and Prevention (KCDCP), and are freely available from KCDCP (https://knhanes.cdc.go.kr, accessed on 9 September 2021).
